# Capillary Triglycerides in Late Pregnancy—Challenging to Measure, Hard to Interpret: A Cohort Study of Practicality

**DOI:** 10.3390/nu13041266

**Published:** 2021-04-13

**Authors:** Helen L. Barrett, Marloes Dekker Nitert, Michael D’Emden, Barbara Lingwood, Susan de Jersey, H. David McIntyre, Leonie K. Callaway

**Affiliations:** 1 Mater Research Institute, The University of Queensland, South Brisbane, QLD 4101, Australia; h.d.mcintyre@uq.edu.au; 2 Department of Endocrinology, Mater Health, South Brisbane, QLD 4101, Australia; 3 School of Chemistry and Molecular Biosciences, The University of Queensland, St Lucia, QLD 4072, Australia; m.dekker@uq.edu.au; 4 The Royal Brisbane and Women’s Hospital, Herston, QLD 4029, Australia; Michael.dEmden@health.qld.gov.au (M.D.); Susan.deJersey@health.qld.gov.au (S.d.J.); Leonie.Callaway@health.qld.gov.au (L.K.C.); 5 School of Medicine, The University of Queensland, Herston, QLD 4006, Australia; 6 UQ Centre for Clinical Research, The University of Queensland, Herston, QLD 4006, Australia; b.lingwood@uq.edu.au; 7 Department of Obstetric Medicine, Mater Health, South Brisbane, QLD 4101, Australia

**Keywords:** capillary, gestational diabetes, mixed meal test, pregnancy, triglycerides

## Abstract

Background: Maternal triglycerides are increasingly recognised as important predictors of infant growth and fat mass. The variability of triglyceride patterns during the day and their relationship to dietary intake in women in late pregnancy have not been explored. This prospective cohort study aimed to examine the utility of monitoring capillary triglycerides in women in late pregnancy. Methods: Twenty-nine women (22 with gestational diabetes (GDM) and 7 without) measured capillary glucose and triglycerides using standard meters at home for four days. On two of those days, they consumed one of two standard isocaloric breakfast meals: a high-fat/low-carbohydrate meal (66% fat) or low fat/high carbohydrate meal (10% fat). Following the standard meals, glucose and triglyceride levels were monitored. Results: Median capillary triglycerides were highly variable between women but did not differ between GDM and normoglycaemic women. There was variability in capillary triglycerides over four days of home monitoring and a difference in incremental area under the curve for capillary triglycerides and glucose between the two standard meals. The high-fat standard meal lowered the incremental area under the curve for capillary glucose (*p* < 0.0001). Fasting (rho 0.66, *p* = 0.0002) and postpradial capillary triglycerides measured at home correlated with venous triglyceride levels. Conclusions: The lack of differences in response to dietary fat intake and the correlation between capillary and venous triglycerides suggest that monitoring of capillary triglycerides before and after meals in pregnancy is unlikely to be useful in the routine clinical practice management of women with gestational diabetes mellitus.

## 1. Introduction

Maternal obesity and diabetes in pregnancy are both associated with fetal overgrowth and adverse pregnancy and neonatal outcomes. There risks relate not only to glycemia but also to other aspects of maternal metabolism, particularly maternal lipids and triglycerides. In early pregnancy, maternal metabolism is anabolic with an increase in maternal fat stores; in late pregnancy, maternal metabolism is catabolic, with increased insulin resistance and peripheral adipose tissue lipolysis resulting in increased maternal lipoprotein concentrations and increased lipoprotein triglyceride content [1]. Currently, in clinical practice managing women with diabetes in pregnancy, we focus on glucose and weight control, but there is evidence that elevated maternal triglycerides are associated with increased fetal growth; increased large for gestational age infants; and other adverse pregnancy outcomes such as preeclampsia and preterm delivery [1].

Fasting maternal triglycerides in early pregnancy correlate more strongly with newborn fat mass (r = 0.67, *p* < 0.001) than does maternal glucose (r = 0.48, *p* < 0.05) [2]. In women with diabetes before pregnancy, high maternal triglycerides in late pregnancy are associated with an increased risk of large for gestational age infants [3]. In women with gestational diabetes, maternal triglyceride levels at the time of standard oral glucose tolerance testing are associated with infant birth weight to a similar extent, independently of the glucose levels measured in the glucose tolerance test. [4,5]. Furthermore, repeated formal oral liquid meal tests administered to women at 14–16 and 26–28 weeks gestation showed that fasting and postprandial triglycerides were elevated in obese compared to normal weight women by 14–16 weeks [6]. In the same study, postprandial triglycerides strongly correlated with infant fat % (*r* = 0.57; *p* < 0.01) whereas glucose did not [6]. Therefore, measuring triglyceride patterns throughout the day could be a useful tool to help predict infants at risk of excess growth. If we are going to consider specifically treating maternal triglyceride levels in diabetes in pregnancy, we first need to decide how to measure them. The mainstay of diabetes management is capillary glucose measurement, and there are now portable machines that are able to measure capillary triglycerides.

Women with diabetes in pregnancy routinely measure their blood glucose concentrations multiple times per day using portable blood glucose meters. The use of continuous glucose monitoring systems is also becoming more widespread and generates a large volume of data on variation in blood glucose levels throughout pregnancy. In contrast, detailed daily data of triglyceride fluctuations throughout gestation are not available for either healthy pregnancies or those affected by diabetes. At present, there is no convenient tool akin to a glucose meter for routine measurement of capillary triglycerides in pregnancy. A possible tool is the Roche Accutrend Plus^®®^ meter with triglyceride strips, which measures the hydrolysis of glycerol in a capillary sample to quantify the concentration of triglycerides. Free fatty acid levels are thus not measured, but to our knowledge, there is no current device that measures capillary free fatty acids in the home setting. Home monitoring of triglycerides using the Roche Accutrend Plus^®®^ meter has been done outside pregnancy but not in pregnancy. We have previously validated the Roche Accutrend Plus^®®^ meter with triglycerides strips in pregnancy [7] and undertaken a pilot study of the home use of this device [8]. These studies showed that the meter could be used at home by pregnant women after sufficient education and support and suggested that capillary triglycerides across the day were variable within and between women.

The aims of the current study were firstly to evaluate the variability of triglyceride levels across four days in women with and without gestational diabetes (GDM) in late pregnancy; secondly, to examine the postprandial capillary triglyceride and glucose response to two pragmatic standard test meals at home (high and low fat); and thirdly, to evaluate the practical use of the triglyceride meter in a larger cohort.

## 2. Materials and Methods

Women with and without GDM at two metropolitan quaternary obstetric hospitals were invited to participate and gave written informed consent. The study was approved by the Human Research Ethics Committee of Royal Brisbane and Women’s Hospital (HREC/15/QRBW/119) and The University of Queensland (2015000847). Women were diagnosed with GDM following usual clinical processes at those clinical institutions—a 75 g Oral Glucose Tolerance Test, performed at 24–28 weeks, using WHO diagnostic criteria [9]. They received usual clinical dietary advice and management as per standard clinic processes. Exclusion criteria included preeclampsia at the time of enrolment, multiple gestation, use of oral glucocortiocosteroids at the time of the study, food allergy to the provided meals, and coeliac disease but not BMI. There was an absence of pregnancy data to base a sample size calculation on; however, other studies of post meal capillary triglycerides outside pregnancy suggest a sample size of 21 to detect a difference between high- and low-fat meals in a population with mild hypertriglyceridemia [10] and 13 in a population with type 2 diabetes mellitus [11].

After enrolment between 34 and 38 weeks gestation, a 30 min training session was undertaken in the use of the triglyceride meter for women with GDM and in the use of the glucose and triglyceride meters for women without GDM. Women monitored capillary glucose (Accu-chek^®®^ Aviva Connect [12]) and capillary triglycerides (Roche Accutrend Plus^®®^ meter, using triglyceride strips [13]) at fasting and two hours after each meal and manually recorded results for four days. Within one week of the four-day study period, fasting venous lipids and glucose were measured with standard clinical laboratory analyses in a certified laboratory. Women were asked to keep their activities or dietary behaviours unaltered. For those women with GDM, the pharmacological management of GDM was not altered during the four days of home monitoring.

Participants consumed one of two supplied isocaloric test breakfast meals varying in carbohydrate and fat content on two of the four mornings. All other meals during the four days of the study were at the participant’s discretion. One supplied meal was a high-fat/low-carbohydrate meal, the other a low-fat/high-carbohydrate meal. The high-fat/low-carbohydrate meal consisted of 14% calories from protein/66% fat/20% carbohydrates (35 g of fat) in the form of a croissant, margarine and cheese. The low-fat/high-carbohydrate meal had 17% calories from protein/10% fat/73% carbohydrates (4.6 g of fat) and consisted of cereal, reduced fat milk, fruit juice and reduced fat yogurt. Women were asked to drink water with both meals. After the standard test meal, the participants measured their capillary glucose and triglycerides at 0, 30 min, 60 min, 120 min and 180 min.

The participants also completed a short questionnaire about the ease of use of the triglyceride meter. The questionnaire on the meter included three questions with tickbox answers across a Likert scale: (1) tell us how easy you found the meter to use: very easy, easy after a few goes, a little hard, very hard or impossible; (2) compared to the glucose meter, using the triglyceride meter was: easier, the same, harder, a lot harder or impossible; (3) how many times did you try to measure your triglycerides and were not able to because of problems with the machine: 1–2, 3–5, 5–10 or 10+? They were also provided opportunity to add free text comments.

### 2.1. Statistical Analyses

Graphpad prism v7.04 was used for analysis. Medians and 95% Confidence Intervals (CI) were used for descriptive data, except where indicated in the results. For all analyses, significance was accepted at the 5% level on two tailed testing. Comparisons between groups for normoglycemic women and those with GDM were undertaken with Mann–Whitney U tests for demographic data.

### 2.2. Analysis of Capillary Glucose and Triglycerides

Capillary glucose and triglycerides over the 4 days fasting, and 2 h post breakfast, post lunch and post dinner were calculated for each woman. This 2 h post breakfast measure includes the 2 h measure from the test meals as well. The incremental area under the curve (incremental AUC) was calculated for the supplied test meals for capillary glucose and triglycerides, using only meals where all five data points were obtained. This incremental AUC was calculated by first adjusting all measures to the baseline measure, which was set at 100%, then calculating the AUC of the curve from the baseline. The incremental AUC capillary glucose and capillary triglycerides for each woman after the high- and low-fat test meals were compared using the paired Wilcoxon rank test. Comparisons between capillary measures and incremental AUC for test meals between GDM and control women were undertaken using an unpaired Mann–Whitney U-test. Correlations between venous measures of glucose and triglyceride and the capillary measures and incremental AUC were undertaken using Spearman’s rho correlation coefficient.

## 3. Results

A total of 29 women completed the study, 22 with GDM and 7 without. In the GDM group, 12 women (55%) were managed with diet and lifestyle advice alone, six women (27%) with insulin, two women (9%) with insulin and metformin and two women (9%) with metformin alone. The pharmacological management of GDM was not altered during the four days of home monitoring. Given the small numbers, we have not analysed the results according to type of pharmacological management for GDM. Demographic data for mother and infant as well as baseline venous blood results are presented in Table 1.

**Figure 1:**
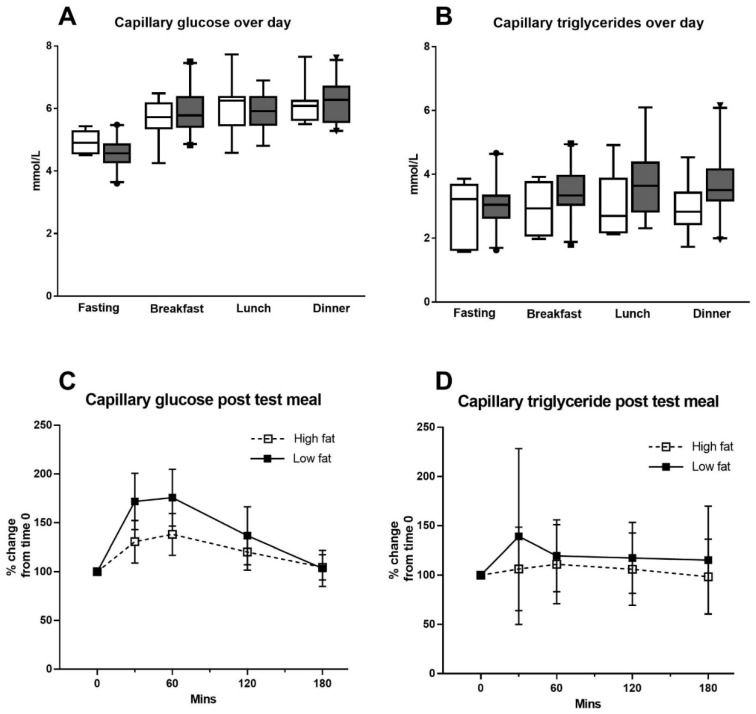
Capillary glucose and triglycerides. Levels averaged over 4 days at fasting, 2 h post breakfast, 2 h post lunch, 2 h post dinner for glucose (**A**) and triglycerides (**B**), respectively, split by GDM status. Control white, GDM grey. Data presented as box plots with 95%CI. (**C**) presents aggregate data for the cohort for glucose response to the high- and low-fat meals, (**D**) presents aggregate data for the cohort for triglycerides response to the high- and low-fat meals; mean and SD.

Figure 1 and Table 2 show the capillary glucose and triglyceride measures across the day. Figure 1 also shows the capillary glucose and triglyceride levels after the supplied test meals. There were no differences between capillary glucose and triglyceride levels for GDM and control women at each time point across the day (Figure 1A,B). Thus, the results of women from both GDM and non-GDM groups were pooled for analysis.

**Figure 2:**
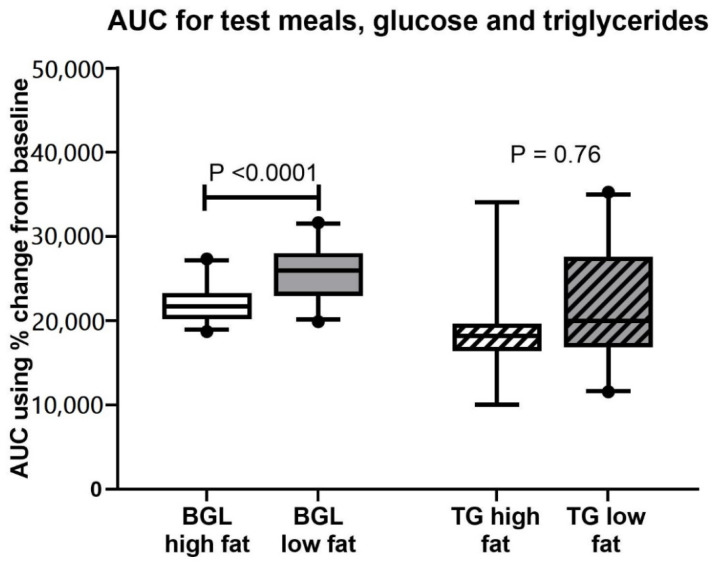
Area under the curve for meals. Figure presents capillary glucose and triglyceride area under the curve, adjusted as % change from baseline with baseline set to 100%, by high- and low-fat meal. 22 complete pairs for glucose, 15 complete pairs for triglycerides. Wilcoxon rank test, paired, Box plot with 95% CI.

For the test meals, 25 of 29 women (86%) completed glucose measures for all five timepoints with the high-fat meal and 23 (79%) for the low-fat meal (Figure 1C). Eighteen women (62%) completed the triglyceride measures for all five timepoints for the high-fat meal and 20 (69%) for the low-fat meal (Figure 1D). There were 22 matched sets of full five timepoint profiles for both test meals for capillary glucose and 15 matched sets of full five timepoint profiles for triglycerides to calculate incremental AUC (calculated as % change from baseline, which was set at 100%) after both test meals, shown in Figure 2. The capillary glucose incremental AUC was significantly lower for the high-fat (21,510 (21,070–23,097) arbitrary units) than the low-fat test meal (25,913 (24,213–27,215) arbitrary units, *p* < 0.0001) (Figure 2). However, the capillary triglyceride incremental AUC was not different between meals (incremental AUC_triglycerides_ high-fat 19,230 (16,477–23,127) arbitrary units vs. incremental AUC_triglycerides_ low-fat 19,095 (17,390–22,480) arbitrary units, *p* = 0.76) (Figure 2). Sensitivity analyses of the incremental AUCs in glucose or triglycerides after the test meals including only women with GDM; only women with diet controlled GDM; and diet-controlled GDM combined with control women showed that all permutations displayed a similar pattern to the full cohort with a significantly lower incremental AUC_glucose_ for the high-fat meal but no difference in incremental AUC_triglycerides_ between meals. The median capillary level for triglyceride and glucose at each timepoint post meal for the group is presented in Table 2.

**Figure 3:**
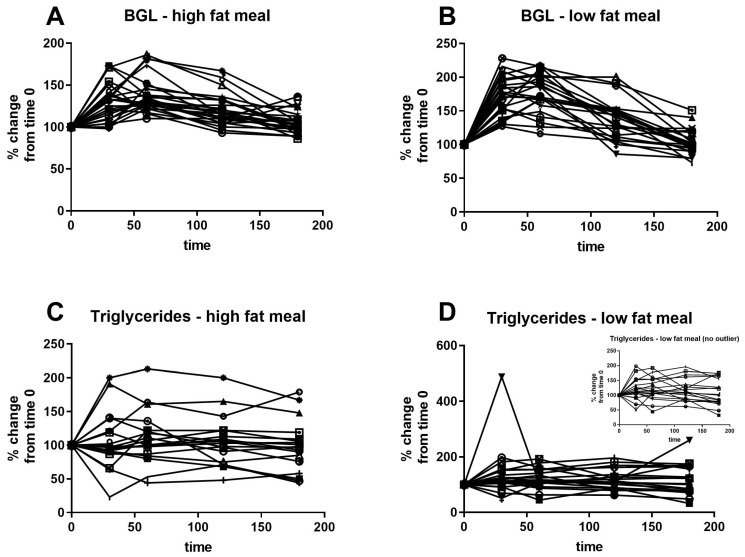
Individual response to high- and low-fat test meals. (**A**,**B**) Capillary glucose, (**C**,**D**) capillary triglycerides. In (**D**), there is one individual with a markedly elevated triglyceride response at time 30 min. The inset graph shows the same data after removal of the data of that individual.

Figure 3 shows the individual response to high- and low-fat meals for capillary glucose and triglycerides, analysed as percentage change from baseline. The inter-individual variability was greater than the intra-individual variability in response to the two test meals for triglycerides. Further, the triglyceride profiles were highly variable, with some individuals displaying a fall in circulating triglycerides in the postprandial period, while others showed a rise in triglycerides, and this was unrelated to GDM status. In contrast, all participants showed a significant rise in glucose concentrations after the meals.

**Figure 4:**
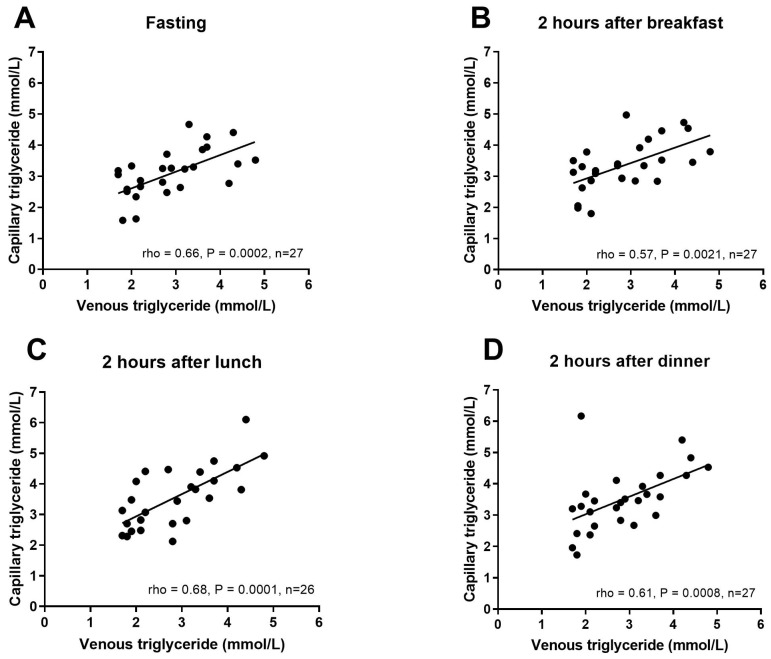
Spearman rho correlation between single fasting venous triglyceride measure and mean of capillary triglyceride measures taken over 4 days. Venous triglycerides versus (**A**) mean of capillary triglycerides at home before breakfast, fasting (**B**) mean of capillary triglycerides at home 2 h after breakfast, (**C**) mean of capillary triglycerides at home 2 h after lunch and (**D**) mean of capillary triglycerides at home 2 h after dinner.

Correlations between laboratory venous triglycerides and average fasting and postprandial triglyceride levels after each meal as measured by the participant at home were determined (Figure 4). Venous triglycerides were moderately and significantly positively correlated with both fasting and postprandial capillary measures of triglycerides. Neither fasting venous glucose nor triglyceride were significantly correlated with the respective incremental AUC measure after either test meal. The fasting and postprandial capillary measures across the day were also correlated with each other, with mean fasting capillary triglycerides being positively correlated with post-breakfast (rho = 0.61, *p* = 0.0006), post-lunch (rho = 0.46, *p* = 0.01) and post-dinner triglycerides (rho = 0.45, *p* = 0.02). Post-breakfast capillary triglycerides were also correlated with post-lunch (rho = 0.60, *p* = 0.001) and post-dinner triglycerides (rho = 0.65, *p* = 0.0002). Lastly, post-lunch and post-dinner capillary triglycerides were also strongly correlated (rho = 0.82, *p* = 0.0001).

Twenty-four of the 29 women provided feedback on the use of the triglyceride meter. Of these, 4 out of 29 found it very hard or impossible and compared it to using a glucose meter, 18 found it harder and 1 found it impossible. Eighteen women had difficulty with measuring triglycerides 1–5 times over the four days, and three had difficulties more than five times over the course of the study. Women commented on the temperature sensitivity of the machine leading to occasional absence of recorded values in both summer and winter, and on the comparatively large volume of blood needed for measurement.

## 4. Discussion

The current study explores the use of a capillary triglyceride meter to monitor maternal triglyceride levels at home in late pregnancy. It also examines late pregnancy capillary glucose and triglyceride responses to a high- and low-fat standard meal. The results suggest that individual maternal capillary triglycerides do not vary greatly across the day or in response to food intake, whereas interindividual variation is high and this is unrelated to GDM status. Formal laboratory measurements of venous triglycerides were highly correlated with measures of capillary triglycerides. Given that short-term food choices do not alter triglyceride results, and that triglyceride readings between participants were widely spread, home monitoring appears to be of limited value.

The differences in triglyceride levels between women were more pronounced than within women. Outside pregnancy, high variability in the fasting and postprandial triglyceride levels in 24 people with type 2 diabetes has also been reported [15]. There are multiple factors that can influence postprandial metabolism, including food intake in the hours preceding the test meal, stress, activity level [16] and the medications used for GDM, none of which were controlled for in the current study. Maternal use of prandial insulin or metformin would suppress glucose response to a meal and influence circulating free fatty acids. As mentioned above, the triglyceride meter used in this study does not measure free fatty acids.

Outside pregnancy, capillary triglycerides have been examined using the same or similar meters. In one study, 39 people with hypertriglyceridemia who received a standard meal (800 kcal, 30% fat) with an additional fat (300 kcal) or carbohydrate supplement (300 kcal) for dinner once a week for three weeks monitored triglycerides with the Roche Accutrend GCT^®®^ meter. It was reported that the 2 and 3 h post-meal triglycerides contained more of the fat supplement than the carbohydrate supplement [10]. Other studies have reported a distinct difference in postprandial triglycerides in people with diabetes compared to controls in the setting of a fat load of 68 g [17]. In 140 adults with type 2 diabetes, triglyceride levels after a lunch meal were examined using the Roche Accutrend GCT^®®^ meter and showed that the postprandial triglyceride levels were positively related to fat intake but inversely to carbohydrate intake measured by questionnaire about average dietary habits [18]. In the current study, we found that there was no difference in incremental AUC triglycerides measured over two hours postprandially between meals, whereas AUC glucose rose reflected the macronutrient content of the meal.

The postprandial response to meals in late pregnancy has previously been investigated. A three day crossover of isocaloric high carbohydrate/low-fat diet (60%CHO/25%fat/15%protein) compared to low carbohydrate/high-fat diet (40% CHO/45%fat/15% protein) in 16 women found no difference in serum triglyceride incremental AUC but lower serum free fatty acids in the high carbohydrate/low-fat diet after breakfast on day 4 of the diet [19]. An isocaloric breakfast meal with equivalent macronutrient composition but different types of fat intake in 18 women in late pregnancy did not report any alterations in the postprandial serum triglyceride levels [20]. Both of these studies attempted to control for overall diet and medication use before and during the meal, but in the setting of a less dramatic difference in dietary composition compared to the studies outside pregnancy described in the above paragraph, no difference in triglycerides was seen. This is similar to our findings.

Taken together, this suggests that the response in triglycerides to a particular meal in late pregnancy could be negligible, unless there is a particularly high fat load. Another factor may be that a large dietary fat load that might influence capillary triglycerides by absorption from the gut lumen might take longer than two hours to show an effect. But for pragmatic reasons, measuring at 1–2 h post meal would be most viable for women monitoring during pregnancy. One aspect to consider would be the total meal “fat load”. In this study, we chose representative samples of common breakfast foods, comparing response to fat load of 35 g versus 3.6 g. This difference may not be sufficient stimulus to provide a detectable clinical difference. The meal fat loads in the studies outside pregnancy described above were more extreme. It does, however, tell us that to detect a difference, the dietary changes may need to be very large. Such dramatic dietary interventions are unlikely to be the mainstay of dietary advice for women with GDM.

The aspects of this study that make analysis complex are the heterogeneous cohort and the uncontrolled nature of the dietary intake apart from the two test meals. This study included both women with and without GDM. Those with gestational diabetes also required different pharmacological treatments, which were not altered during the study. The sensitivity analyses showed that capillary glucose was lower with the low-carbohydrate test meal in all groups of women and that no group displayed differences in the capillary triglycerides after the high- and low-fat meals. The limited number of women enrolled in the current study who did not have GDM is reflective of the burden this study placed on women who participated, which was more noticeable for women without GDM who were not used to monitoring their glucose levels at home already.

The strength of this study is that we have, over a series of investigations, validated the Roche Accutrend Plus^®®^ meter for use in late pregnancy [7], trialled it in a small group in the home setting [8] and now examined postprandial triglycerides in a larger group, with a pragmatic and authentic meal test to assess whether differences in a standard meal response can be detected using this meter. We once again found that the meter is more difficult to use than a glucose meter.

The current study shows that the measurement of maternal capillary triglycerides in late pregnancy is possible. However, the large between-person response in capillary triglycerides unrelated to GDM status overshadows the small within-person response to the high- and low-fat test meals. There does not seem to be the same predictable postprandial “curve” of capillary triglycerides compared to capillary glucose measures that guide dietary management. A larger study would be needed to examine the variability in the postprandial curves. Such a study might benefit from tighter control of experimental conditions, but this would be at the expense of “real world” generalisability. When home monitoring is used for glucose, there is a clear difference in glucose with different carbohydrate intakes for meals that provide biofeedback and guide dietary counselling and advice in clinical settings. Triglyceride monitoring in this study did not provide relevant feedback that might be similarly useful to guide clinical advice.

The current study suggests that home measurement of capillary triglycerides at multiple time points does not show promise as a way to improve clinical care of women with gestational diabetes mellitus.

## Figures and Tables

**Table 1:** Maternal and infant demographic details. These data are for the whole group, gestational diabetes (GDM) and normoglycemic women combined ^a^.

	*n*	Total Cohort (*n* = 29)	GDM (*n* = 22)	Normoglycemia (*n* = 7)	*p*
Maternal age at delivery, years	29	33 (30.6–36.1)	33 (30.6–36.2)	32 (29.5–38.2)	0.64
Gestational age of enrolment, days	29	252 (243–254)	253 (242–255)	250 (214–267)	0.72
G, *n* (%)	29	2 (2–3)	2 (1–3)	2 (1–4)	
P, *n* (%)	29	1 (0–1)	1 (0–1)	0 (0–3)	
BMI—recalled prepregnancy weight, kg/m^2^	28	25.1 (21.3–28.3)	25.3 (20.9–29)	23.6 (18–29.3)	0.64
BMI—enrolment, kg/m^2^	27	29.7 (26.0–30.7)	28.8 (26–30.7)	29.8 (23.1–33.9)	0.8
SBP enrolment, mmHg	28	109 (100–118)	108 (100–120)	110 (98–119)	0.77
DBP enrolment, mmHg	28	70 (60–72)	70 (60–75)	70 (58–78)	0.91
Caucasian ethnicity, *n* (%)	29	20 (68.9)	16 (72.7)	4 (57.1)	
Diagnosis of GDM, *n* (%)	29	22 (75.8)	22 (100)	-	
Management of GDM with diet/exercise alone, *n* (%)	22	12 (55)	12 (55)	-	
Management of GDM with insulin alone, *n* (%)	22	6 (27)	6 (27)	-	
Management of GDM with metformin alone, *n* (%)	22	2 (9)	2 (9)	-	
Vaginal delivery, *n* (%)	29	18 (62)	14 (63.6)	4 (57.1)	
**Fasting Maternal Bloods**					
HbA1c mmol/mol	25	32 (30–34)	32 (30–34)	32 (29–36)	0.97
HbA1c, %	25	5.1 (4.9–5.3)	5.1 (4.9–5.3)	5.1 (4.76–5.4)	0.97
Glucose, mmol/L	27	4.3 (4–4.7)	4.5 (4.2–4.8)	3.8 (3.4–4.5)	0.01
HDL mmol/L	27	1.7 (1.5–1.9)	1.7 (1.5–1.8)	2.0 (1.5–2.4)	0.21
LDL, mmol/L	27	3.5 (2.6–4.2)	3.5 (2.5–4.4)	4.1 (2.3–5.8)	0.56
Triglyceride, mmol/L	27	2.8 (2.1–3.4)	2.7 (2.1–3.3)	3.2 (1.8–4.8)	0.66
Total Cholesterol, mmol/L	27	6.9 (5.5–7.7)	6.8 (5–7.7)	8.2 (53)	0.26
**Infant Parameters**					
Gestational age of delivery, days	29	274 (268–278)	274 (267–278)	276 (255–285)	0.57
Birth weight, g	29	3450 (3174–3645)	3435 (3140–3760)	3450 (2909–4145)	0.74
Birth weight centile ^b^, %	29	37 (24.4–70.3)	36 (32–58)	55 (28–80)	0.41
Birth length, cm	29	51 (49.5–52.5)	51 (49–53)	51 (49.5–52.5)	>0.99
Birth weight >4000 g, *n* (%)	29	3 (10.3%)	2 (9)	1 (14.2)	
Female, *n* (%)	29	17 (58.6)	14 (63.6)	3 (42.9)	
NICU admission, *n* (%)	29	2 (6.8)	1 (4.5)	1 (14.2)	
SCN admission, *n* (%)	29	3 (10.3)	0	3 (42.9)	

^a^ Data is presented as median and 95% Confidence Intervals (CI) unless otherwise indicated. ^b^ Centile calculated from the Gestation Network centile calculator, which adjusts for infant sex, gestational age of delivery, maternal Body Mass Index (BMI), ethnicity and parity [14]. NICU—neonatal intensive care unit, SCN—special care nursery.

**Table 2:** Measures of capillary glucose and triglycerides.

Capillary Measures	
**Glucose, mmol/L**	
Fasting	4.6 (4.4–4.9), (3.6–5.5)
2 h post breakfast	5.7 (5.4–6.1), (4.3–7.5)
2 h post lunch	6.1 (5.6–6.3), (4.6–7.7)
2 h post dinner	6.2 (5.6–6.5), (5.3–7.7)
**Triglycerides, mmol/L**	
Fasting	3.1 (3.1–3.5), (1.6–4.7)
2 h post breakfast	3.3 (2.9–3.8), (1.8–5)
2 h post lunch	3.5 (2.7–4.1), (2.1–6.1)
2 h post dinner	3.4 (3–3.7), (1.7–6.2)

Data expressed as median (95%CI), (range).

## Data Availability

Datasets generated during and/or analyzed during the current study are not publicly available but are available from the corresponding author on reasonable request.
